# The ketogenic diet preserves skeletal muscle with aging in mice

**DOI:** 10.1111/acel.13322

**Published:** 2021-03-06

**Authors:** Marita A. Wallace, Nicholas W. Aguirre, George R. Marcotte, Andrea G. Marshall, Leslie M. Baehr, David C. Hughes, Karyn L. Hamilton, Megan N. Roberts, Jose Alberto Lopez‐Dominguez, Benjamin F. Miller, Jon J. Ramsey, Keith Baar

**Affiliations:** ^1^ Department of Neurobiology, Physiology and Behavior University of California Davis CA USA; ^2^ CellMet Performance Health Perth WA Australia; ^3^ Department of Health and Exercise Science Colorado State University Fort Collins CO USA; ^4^ Department of Molecular Biosciences School of Veterinary Medicine University of California Davis CA USA; ^5^ Aging and Metabolism Research Program Oklahoma Medical Research Foundation Oklahoma City OK USA; ^6^ Department of Physiology and Membrane Biology School of Medicine University of California Davis CA USA

**Keywords:** aging, ketogenic diet, mice, sarcopenia, skeletal muscle

## Abstract

The causes of the decline in skeletal muscle mass and function with age, known as sarcopenia, are poorly understood. Nutrition (calorie restriction) interventions impact many cellular processes and increase lifespan and preserve muscle mass and function with age. As we previously observed an increase in life span and muscle function in aging mice on a ketogenic diet (KD), we aimed to investigate the effect of a KD on the maintenance of skeletal muscle mass with age and the potential molecular mechanisms of this action. Twelve‐month‐old mice were assigned to an isocaloric control or KD until 16 or 26 months of age, at which time skeletal muscle was collected for evaluating mass, morphology, and biochemical properties. Skeletal muscle mass was significantly greater at 26 months in the gastrocnemius of mice on the KD. This result in KD mice was associated with a shift in fiber type from type IIb to IIa fibers and a range of molecular parameters including increased markers of NMJ remodeling, mitochondrial biogenesis, oxidative metabolism, and antioxidant capacity, while decreasing endoplasmic reticulum (ER) stress, protein synthesis, and proteasome activity. Overall, this study shows the effectiveness of a long‐term KD in mitigating sarcopenia. The diet preferentially preserved oxidative muscle fibers and improved mitochondrial and antioxidant capacity. These adaptations may result in a healthier cellular environment, decreasing oxidative and ER stress resulting in less protein turnover. These shifts allow mice to better maintain muscle mass and function with age.

## Graphical abstract

Nutrition interventions impact the preservation of skeletal muscle mass and function with age. This study demonstrated that the ketogenic diet (KD) improved skeletal muscle mass preservation with age. As muscle progresses from adult to old, individuals on a standard control (CON) diet show less reinnervation and more unfolded proteins. By contrast, individuals on a long‐term KD showed more mitochondria, greater reinnervation, more oxidative muscle fibers, and decreased translation initiation.

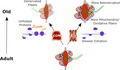

## 1 INTRODUCTION

Sarcopenia is the age‐related loss of muscle mass and function that occurs in the absence of disease (Larsson et al., 2019). The incidence of sarcopenia is reported to range from 14% in 65–69 year‐olds to 50% in the 80+ population (Janssen, 2010). Sarcopenia reduces the ability to perform activities of daily living and contributes to a loss of mobility and independence, as well as an increased risk of frailty, falls, morbidity and mortality. The loss of muscle mass with aging results from a decrease in both muscle fiber number and fiber cross‐sectional area (Larsson et al., 2019). However, the exact processes that underpin the development of sarcopenia are not completely understood. Several mechanisms have been proposed to explain sarcopenia including: neuromuscular junction instability (Baehr et al., 2016; Rudolf et al., 2014), mitochondrial dysfunction (Coen et al., 2018), endoplasmic reticulum (ER) stress (Baehr et al., 2016; Deldicque, 2013), oxidative stress (Deepa et al., 2019; Sullivan‐Gunn & Lewandowski, 2013), inflammation (Nelke et al., 2019; Peake et al., 2010), anabolic resistance (Breen & Phillips, 2011), protein malnutrition (Phillips & Martinson, 2019; Robinson et al., 2018), and the dysregulation of proteostasis (Murton, 2015). As the global population is aging rapidly, it is critical to uncover the mechanisms responsible for the loss of muscle with aging and develop strategies to slow the progression of or prevent sarcopenia.

Sarcopenia can be greatly accelerated by lifestyle choices, such as physical inactivity and poor nutrition (Larsson et al., 2019). Nutrition is widely recognized as an effective intervention to reduce the loss of muscle mass and function and prolong independence and quality of life. Calorie restriction (CR) without malnutrition is considered one of the most powerful anti‐aging interventions and has been shown to extend both mean and maximum lifespan in multiple species (Redman & Ravussin, 2011). In skeletal muscle, CR delays the onset and slows the progression of sarcopenia (Marzetti et al., 2009). The exact mechanisms as to how CR preserves muscle mass with aging are still unclear; however, the shift from carbohydrate to fat metabolism and increased ketone levels is one mechanism hypothesized to underlie these benefits (Rhoads et al., 2020).

Protein restriction also can lead to prolongation of lifespan in model organisms and decreases mortality in people under 65 years old (Levine et al., 2014). Unlike caloric restriction, protein restriction results in a conserved decrease in insulin‐like growth factor (IGF)‐1 levels (Levine et al., 2014) and the activity of the IGF‐1/akt/mechanistic target of rapamycin complex 1 (mTORC1) pathway (Wei et al., 2009) leading to a decrease in tumor growth (Fontana et al., 2013). However, a prolonged decrease in dietary protein may result in the acceleration of sarcopenia (the age‐dependent loss of muscle mass in the absence of disease) in humans (Phillips & Martinson, 2019; Robinson et al., 2018). In fact, older men need greater dietary protein levels to achieve the same amount of muscle protein synthesis (Moore et al., 2015) leading many to suggest that increased protein intake may be required in older humans to prolong lifespan (Ruiz et al., 2008).

Very low carbohydrate or ketogenic diets (KD) may provide the effects of both caloric restriction, shifting metabolism from carbohydrates toward fatty acids, and protein restriction, decreasing global AKT/mTORC1 signaling. Consequently, we (Roberts et al., 2018) and others (Newman et al., 2017) have shown that a KD increases life and health span in mice, while the ketone, β‐hydroxybutyrate (BHB), increases life span in *C. elegans* (Edwards et al., 2014). In addition to its traditional role in energy metabolism, BHB has also been shown to act as a signaling molecule (Miller et al., 2018). Mechanistically, the KD or ketone bodies have been suggested to be neuroprotective (Maalouf et al., 2009), improve mitochondrial content and function (Miller et al., 2018), reduce insulin/insulin‐like growth factor (IGF‐1) signaling (McDaniel et al., 2011), activate autophagy (Wang et al., 2018), increase antioxidants (Milder & Patel, 2012), and have anti‐inflammatory effects (Dupuis et al., 2015); all of which may influence skeletal muscle aging.

We recently observed a preservation of motor function in aging mice consuming a KD compared to a standard control diet (Roberts et al., 2018). This preservation of motor function was associated with higher relative weights of several hind limb muscles in old mice on the KD. In humans, the KD has a protective effect on muscle mass compared to a low‐fat diet during weight loss in the absence of exercise (Wood et al., 2012). There is currently limited knowledge about the effect of the KD on cellular mechanisms in skeletal muscle, although prior investigations in young rodents have demonstrated a rise in markers of mitochondrial content and biogenesis (Hyatt et al., 2016; Parry et al., 2018), an increase in antioxidant protein expression (Hyatt et al., 2016) and a decrease in young (5 months) but an increase in old (28 months) rats in relation to anabolic signaling through the mTOR pathway (Bennett et al., 2019). How long‐term adaptation to the KD, at a molecular level, affects aging skeletal muscle remains unknown.

The present study extended our previous work to compare the effect of isocaloric standard control (CON) or ketogenic diet (KD) initiated in mid‐life (12 months of age) on skeletal muscle of male C57BL/6 J mice. Muscles from mice maintained on a control or ketogenic diet for 14 months in our original study were compared with a new group of animals who were on the diets for 4 months. We compared the effects of diet and age on muscle phenotype, as well as a range of molecular parameters, including those involved in neuromuscular junction (NMJ) plasticity, mitochondrial biogenesis, oxidative metabolism, cellular stress (ER and oxidative stress, inflammation), and protein turnover, were determined. We hypothesized that a KD would improve innervation and mitochondrial function while decreasing inflammation resulting overall better muscle health.

## 2 RESULTS

### 2.1 Skeletal muscle weight and fiber size

**FIGURE 1:**
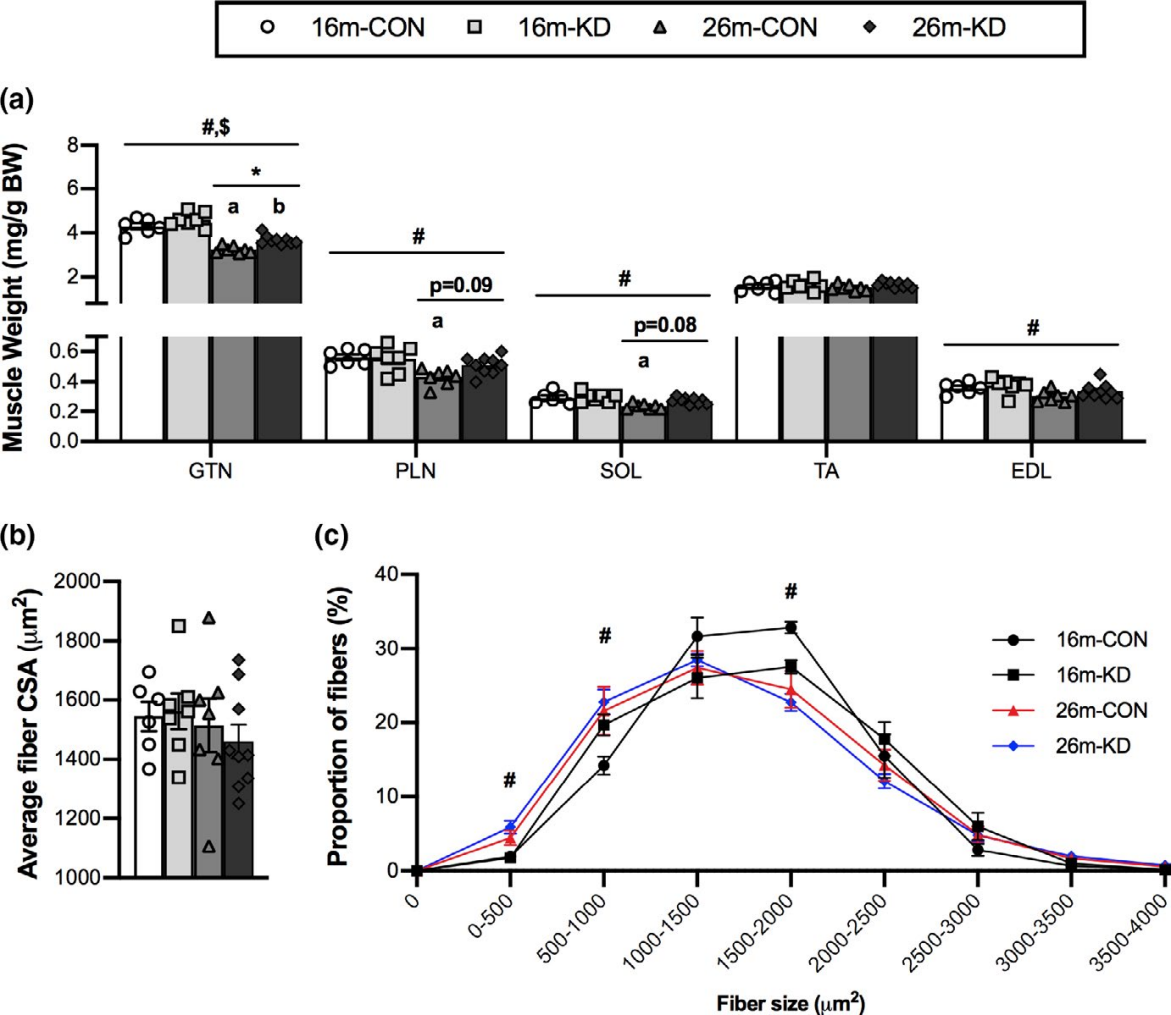
Effect of diet and aging on skeletal muscle weight and fiber size. (a) Gastrocnemius (GTN), plantaris (PLN), soleus (SOL), tibialis anterior (TA), and extensor digitorum longus (EDL) muscle weight relative to body weight (BW) of 16‐ and 26‐month‐old mice on a control (CON) or ketogenic diet (KD). (b) Average fiber cross‐sectional area (CSA) and (c) fiber size distribution from the GTN of 16‐ and 26‐month‐old mice on CON or KD. Values are expressed as means ± SEM. *n* = 6 (16‐month CON), *n* = 7 (16‐month KD and 26‐month CON), and *n* = 9 (26‐month KD). (#) main effect of age. ($) main effect of diet. (*p < 0.05) comparing CON and KD at 16 and 26 months. (a) *p* < 0.05 comparing CON at 16 and 26 months. (b) *p* < 0.05 comparing KD at 16 and 26 months

To determine the impact of diet and age on muscle mass, body weight and wet muscle weights of the gastrocnemius (GTN), plantaris (PLN), soleus (SOL), tibialis anterior (TA), and the extensor digitorum longus (EDL) were measured in animals on isocaloric CON and KD at 16 and 26 months old, representing 4 and 14 months on the diet, respectively. No differences in body weight were observed with age or diet (data not shown). There was a main effect of age in that all relative muscle weights decreased with age, except for the TA (Figure 1a). For the GTN, there was a significant decrease in muscle weight from 16 to 26 months for both diets; however, mice on the KD showed a significantly greater GTN muscle mass at 26 months compared to control diet. For the PLN and SOL, there was a significantly lower muscle mass at 26 compared with 16 months only for the CON diet. There was a trend for higher PLN and SOL muscle weights in the 26‐month‐old mice on the KD compared with the 26‐month‐old CON.

To determine the impact of diet and age on muscle fiber size, we assessed the average fiber cross‐sectional area (CSA) and fiber size distribution in the GTN. There was no effect of age or diet on the average fiber CSA (Figure 1b). The fiber size distribution showed a shift of the distribution curves to the left (smaller) with age, regardless of diet, with a main effect of age resulting in an increase in the percentage of fibers with CSA below 0–1,000 μm^2^ and a decrease in the proportion of fibers with a CSA between 1,500 and 2,000 μm^2^ (Figure 1c).

### 2.2 Skeletal muscle phenotype and neuromuscular junction remodeling

**FIGURE 2:**
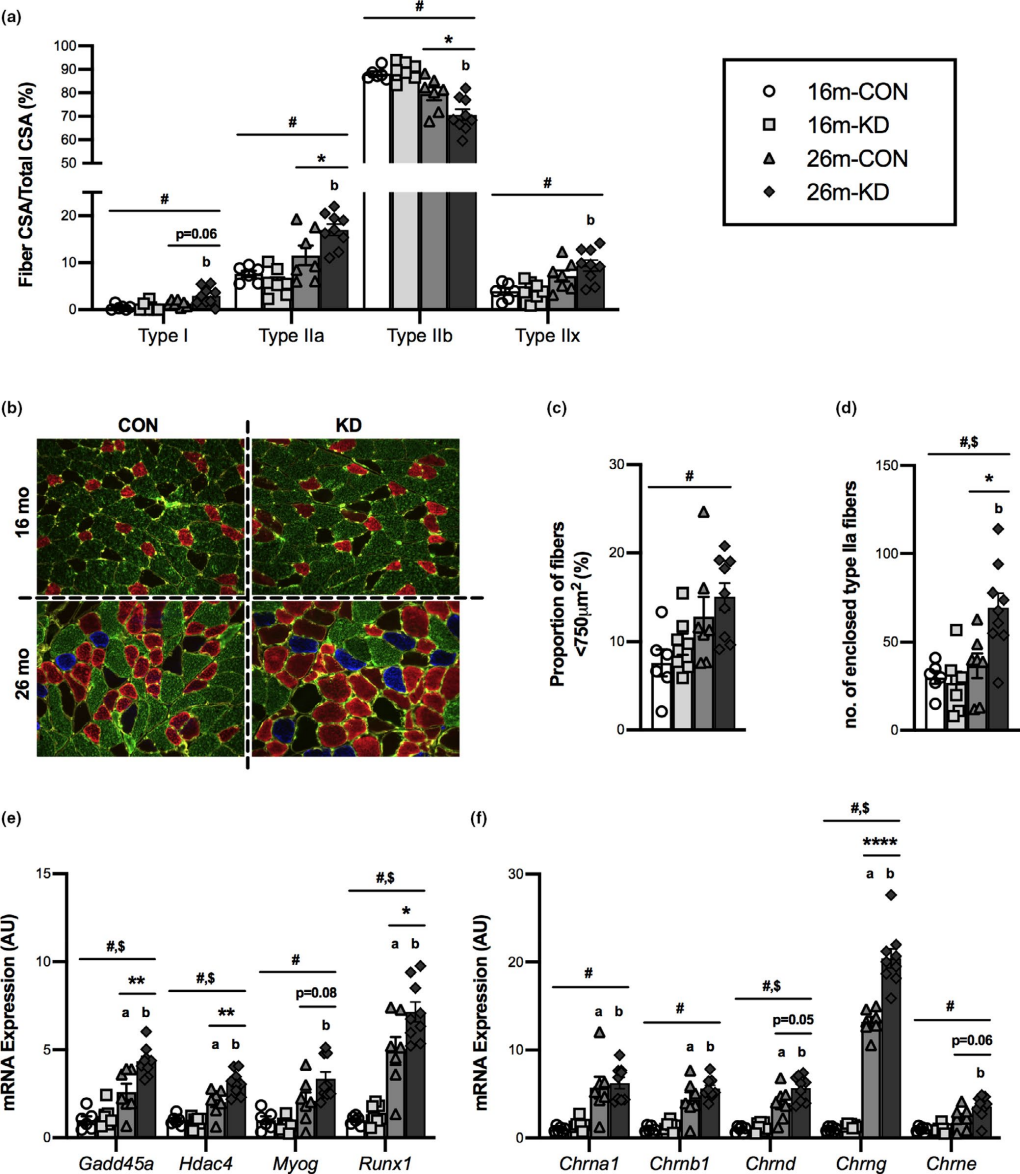
Effect of diet and aging on skeletal muscle phenotype and neuromuscular junction remodeling. (a) Fiber cross‐sectional area (CSA) relative to total fiber CSA for Type I, IIa, IIb, and IIx fibers from the GTN of 16‐ and 26‐month‐old mice on a control (CON) or ketogenic diet (KD). (b) Representative cross‐sectional images (10x objective) from the deep portion of the gastrocnemius of 16‐ and 26‐month‐old mice on the CON or KD. Staining was for Type I (blue), IIa (red), IIb (green), IIx (no stain, black), and laminin (yellow). (c) Proportion of small fibers (<750 μm), (d) total number of enclosed type IIa fibers and mRNA expression of (e) markers of skeletal muscle denervation and (f) acetylcholine receptor subunits from the GTN of 16‐ and 26‐month‐old mice on the CON or KD. Values are expressed as means ± SEM. *n* = 6 (16‐month CON), *n* = 7 (16‐month KD and 26‐month CON), and *n* = 9 (26‐month KD). (#) main effect of age. ($) main effect of diet. (*p < 0.05; **p < 0.01; ****p < 0.0001) comparing CON and KD at 16 and 26 months. (a) *p* < 0.05 comparing CON at 16 and 26 months. (b) *p* < 0.05 comparing KD at 16 and 26 months

To determine the impact of diet and age on muscle phenotype, we compared fiber‐type‐specific CSA between CON and KD at 16 and 26 months (Figure 2a). There was a main effect of age on fiber type with a significant decrease in the proportion of type IIb and a significant increase in the proportion of type I, IIa, and IIx fibers with age. The GTN muscle remained predominately comprised of type IIb fibers regardless of diet or age; however, only animals on the KD showed a significant decrease in the percentage of the muscle composed of type IIb fibers at 26 compared with 16 months old. At 26 months, the percentage of muscle that was comprised of type IIb fibers was significantly higher in CON (80%) when compared to KD (71%). The CON diet resulted in no significant changes in fiber‐type‐specific CSA from 16 to 26 months. Conversely, a significantly greater percentage of type I, IIa, and IIx CSA was observed for the KD at 26 months compared with 16 months. At 26 months, the percentage of muscle that was comprised of type IIa fibers was significantly higher in KD (17%) when compared to CON (12%). Additionally, there was a trend for the percentage of type I fibers to be higher in KD when compared to CON at 26 months. From visual observations of the whole GTN muscles, the changes in fiber type were localized to the deep portion of the GTN, adjacent to the SOL and PLN muscles (Figure 2b).

To determine the impact of diet and age on markers of neuromuscular junction remodeling, we analyzed the presence of small (<750 μm^2^) fibers, measured type IIa fiber clustering and expression of denervation markers including the acetylcholine receptor subunits. There was a main effect of age on the proportion of small fibers (<750 μm^2^) with age showing a higher proportion of small fibers; however, there was no effect of diet (Figure 2c). Type IIa fiber clustering was measured, as a surrogate for axonal sprouting following denervation, by counting the number of type IIa fibers that were enclosed by two or more other type IIa fibers (Figure 2d). There was a main effect of age and diet on the number of enclosed type IIa fibers. Both age and the KD showed a greater number of enclosed type IIa fibers. The number of enclosed type IIa fibers was significantly higher for KD when compared to CON at 26 months. There was a main effect of age on molecular markers of denervation in that the expression of *Gadd45a*, *Hdac4*, *Myog*, and *Runx1* increased with age (Figure 2e). There was also a main effect of diet, with mice on the KD showing greater expression of *Gadd45a*, *Hdac4*, and *Runx1*. The expression of *Gadd45a*, *Hdac4*, and *Runx1* was higher at 26 months than at 16 months for both diets; however, expression was significantly higher for the KD when compared to CON at 26 months. There was also a trend for higher *Myog* expression for the KD when compared to CON at 26 months. There was also a main effect of age on acetylcholine receptor subunits, showing greater expression of all subunits with age (Figure 2f). There was a main effect of diet on *Chrnd* and *Chrng* expression, with the KD showing more of these subunits. The expression of acetylcholine receptor subunits, *Chrna1*, *Chrnb1*, *Chrnd*, and *Chrng*, was significantly higher at 26 compared with 16 months for both CON and KD. The greater *Chrng* expression was higher still with the KD, while there was a trend for greater *Chrnd* and *Chrne* expression on a KD when compared to CON at 26 months. These data suggest that neuronal remodeling increases with age and may be amplified by a KD.

### 2.3 Mitochondrial biogenesis and oxidative metabolism

**FIGURE 3:**
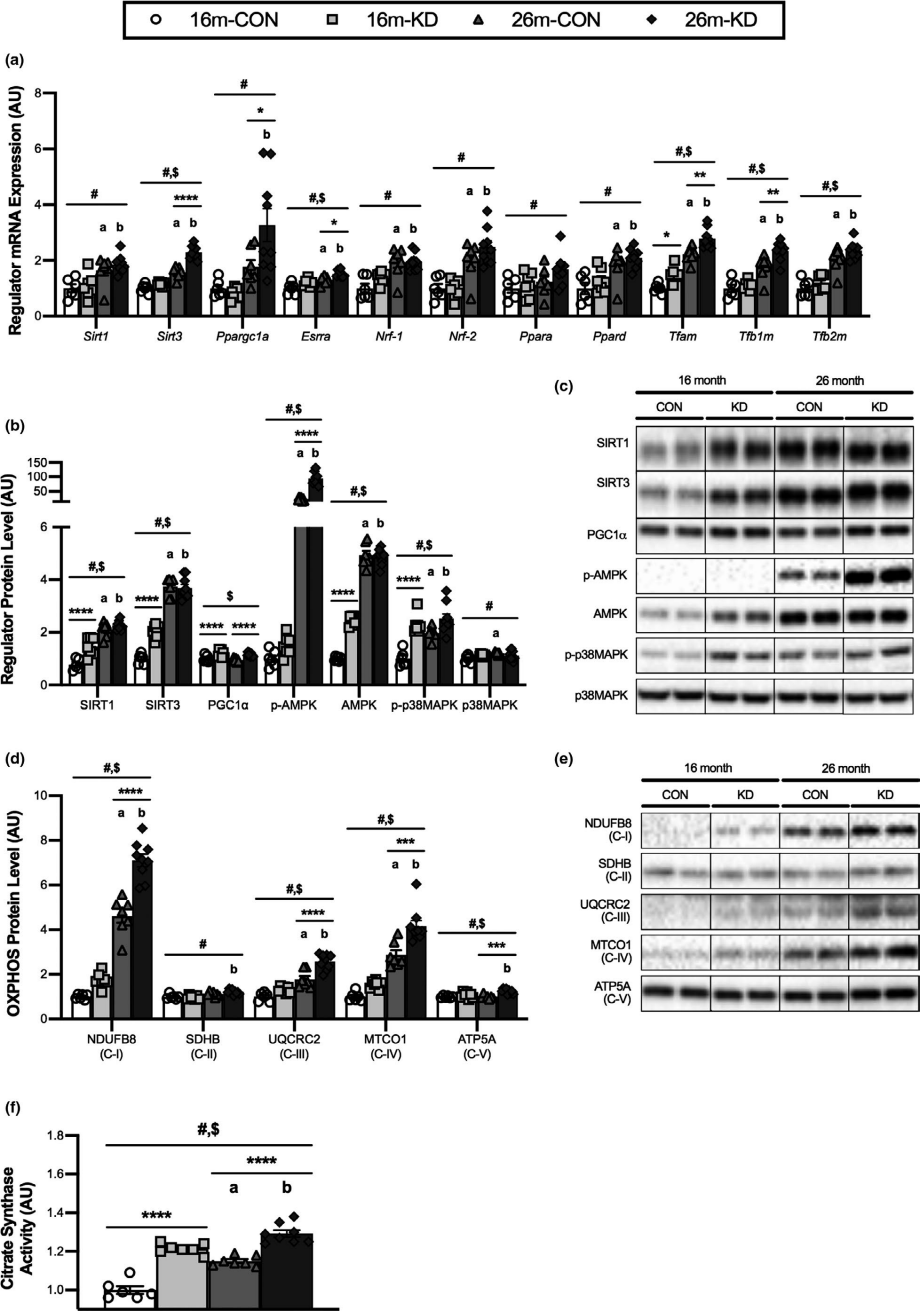
Effect of diet and aging on mitochondrial biogenesis and oxidative metabolism. (a) mRNA expression of transcriptional regulators of mitochondrial biogenesis, protein levels, and representative western blot images of (b,c) regulators of mitochondrial biogenesis, (d,e) oxidative phosphorylation (OXPHOS) proteins from each complex (C‐I to C‐V) and (f) citrate synthase enzymatic activity from the GTN of 16‐ and 26‐month‐old mice on a control (CON) or ketogenic diet (KD). For complete western blot images refer to Appendix S1 (Figure S1). Values are expressed as means ± SEM. *n* = 6 (16‐month CON), *n* = 7 (16‐month KD and 26‐month CON), and *n* = 9 (26‐month KD). (#) main effect of age. ($) main effect of diet. (*p < 0.05; **p < 0.01; ***p < 0.001; ****p < 0.0001) comparing CON and KD at 16 and 26 months. (a) *p* < 0.05 comparing CON at 16 and 26 months. (b) *p* < 0.05 comparing KD at 16 and 26 months

To determine the effect of diet and age on mitochondrial biogenesis, we investigated the expression and levels of several transcriptional regulators of mitochondrial biogenesis in the GTN muscle. There was a main effect of age for the expression of all measured regulators of mitochondrial biogenesis in that the expression of these regulators increased with age (Figure 3a). There was, however, a main effect of diet for only *Sirt3*, *Esrra*, *Tfam*, *Tfb1 m*, and *Tfb2 m* with expression of these regulators being higher in the KD. The expression of *Sirt3*, *Ppargc1a*, *Esrra*, *Tfam*, and *Tfb1 m* was significantly higher with the KD when compared to CON at 26 months. The expression of *Tfam* was also higher in KD group when compared to CON at 16 months.

There was a main effect of age on SIRT1, SIRT3, p‐AMPK, AMPK, p‐p38MAPK, and p38MAPK protein in that levels of these regulators increased with age (Figure 3b,c). For SIRT1, SIRT3, PGC1α, p‐AMPK, AMPK, and p‐p38MAPK levels, there was also a main effect of diet, with all these proteins being further elevated by the KD. The level of SIRT1 and SIRT3 was significantly higher in the KD group when compared to CON at 16 months; however, no effect of diet was observed at 26 months. For PGC1α protein, we observed a significant increase with the KD compared to CON at both 16 and 26 months. Finally, the level of total AMPK and p‐p38MAPK at 16 months and p‐AMPK at 26 months was significantly elevated with the KD when compared to CON. Interestingly, even with the increased level of markers of mitochondrial biogenesis the rate of mitochondrial protein synthesis was lower in the KD compared to CON in 16‐month‐old mice (Figure 5a).

To determine the effect of diet and age on oxidative metabolism we investigated the activity and level of key metabolic enzymes. The level of several proteins within the electron transfer chain (oxidative phosphorylation; OXPHOS), including NDUFB8, UQCRC2, MTCO1, and ATP5A (from complexes I, III, IV, and V, respectively) was significantly higher in the KD group when compared to CON at 26 months (Figure 3d–e). We also observed an increase in NDUFB8, UQCRC2, and MTCO1 levels with both diets and ATP5A for KD only between 16 to 26 months. For the complex II protein, SDHB, we only detected a significant increase from 16 to 26 months for KD (Figure 5b,c). Consistent with the protein data, the activity of the key Kreb's Cycle enzyme, citrate synthase, was significantly higher in the KD group when compared to CON at both 16 and 26 months (Figure 3f). Citrate synthase activity also increased from 16 to 26 months for both diets. These data suggest that mitochondrial mass and function increase between 16 and 26 months of age in GTN and that this increase is amplified by a KD.

### 2.4 Cellular stress responses

**FIGURE 4:**
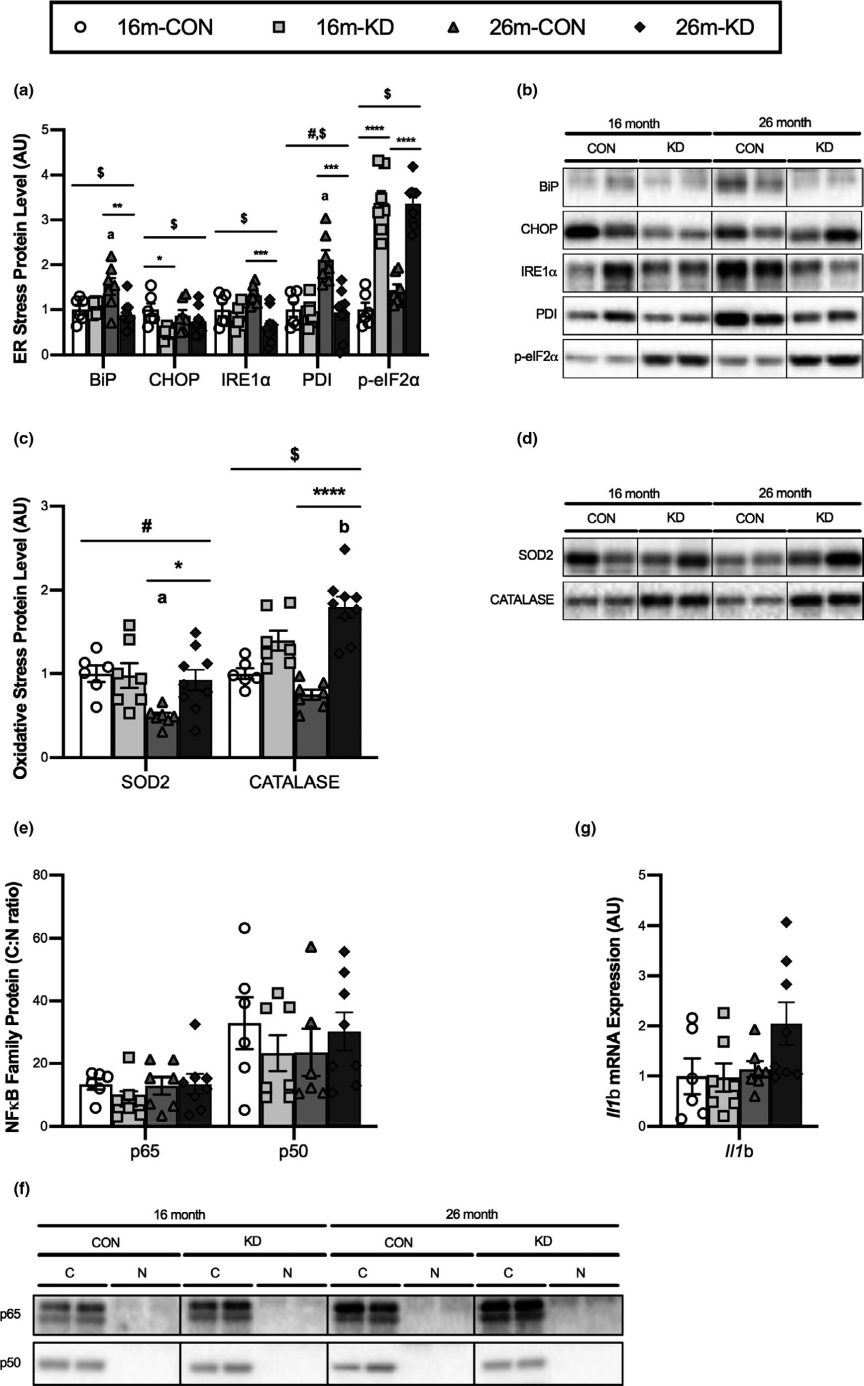
Effect of diet and aging on cellular stress responses. Protein levels and representative western blot images of (a,b) endoplasmic reticulum (ER) stress and (c,d) oxidative stress response proteins from the GTN of 16‐ and 26‐month‐old mice on a control (CON) or ketogenic diet (KD). For complete western blot images, refer to Appendix S1 (Figure S2). (e) Ratio of cytoplasmic to nuclear (C:N) proteins for conical NFκB signaling family members and (f) their representative western blot images from the quadricep of 16‐ and 26‐month‐old mice on a CON or KD. For complete western blot images refer to Appendix S1 (Figure S3). (g) *Il1b* mRNA expression in the GTN of 16‐ and 26‐month‐old mice on the CON or KD. Values are expressed as means ± SEM. *n* = 6 (16‐month CON), *n* = 7 (16‐month KD and 26‐month CON), and *n* = 9 (26‐month KD). (#) main effect of age. ($) main effect of diet. (*p < 0.05; **p < 0.01; ***p < 0.001; ****p < 0.0001) comparing CON and KD at 16 and 26 months. (a) *p* < 0.05 comparing CON at 16 and 26 months. (b) *p* < 0.05 comparing KD at 16 and 26 months

To determine the effect of diet and age on the ER stress response, we measured the level of key ER stress proteins (Figure 4a,b). There was a main effect of diet on all measured proteins, with BiP, CHOP, IRE1α, and PDI levels being decreased and p‐eIF2α levels being increased with the KD. BiP protein increased significantly between 16 and 26 months on the CON diet, whereas on the KD, the increase in BiP did not occur. At 16 months, the maladaptive response protein CHOP was significantly lower on the KD when compared to CON; however, there were no differences at 26 months. Levels of IRE1α and PDI were significantly lower on the KD at 26 months. Conversely, the phosphorylation of eIF2α (p‐eIF2α) was significantly increased in the KD group when compared to CON at both 16 and 26 months.

To determine the effect of diet and age on antioxidant capacity, we measured the levels of two antioxidant proteins, SOD2 and catalase (Figure 4c,d). At 26 months, both SOD2 and catalase were significantly higher in the KD group when compared to CON. From 16 to 26 months, there was a significant decrease in SOD2 on the CON diet, whereas there was an increase in the level of catalase on a KD. To determine the impact of diet and age on inflammatory signaling within muscle, we determined the cellular localization of canonical NFκB signaling family members and the expression of the NFκB transcriptional target, IL‐1β in the quadricep (QUAD) muscle. There was no effect of age or diet on the cytoplasmic to nuclear protein ratio of p65 or p50 (Figure 4e,f). Additionally, there was no effect of age or diet on *il1b* expression (Figure 4g).

### 2.5 Proteostasis

**FIGURE 5:**
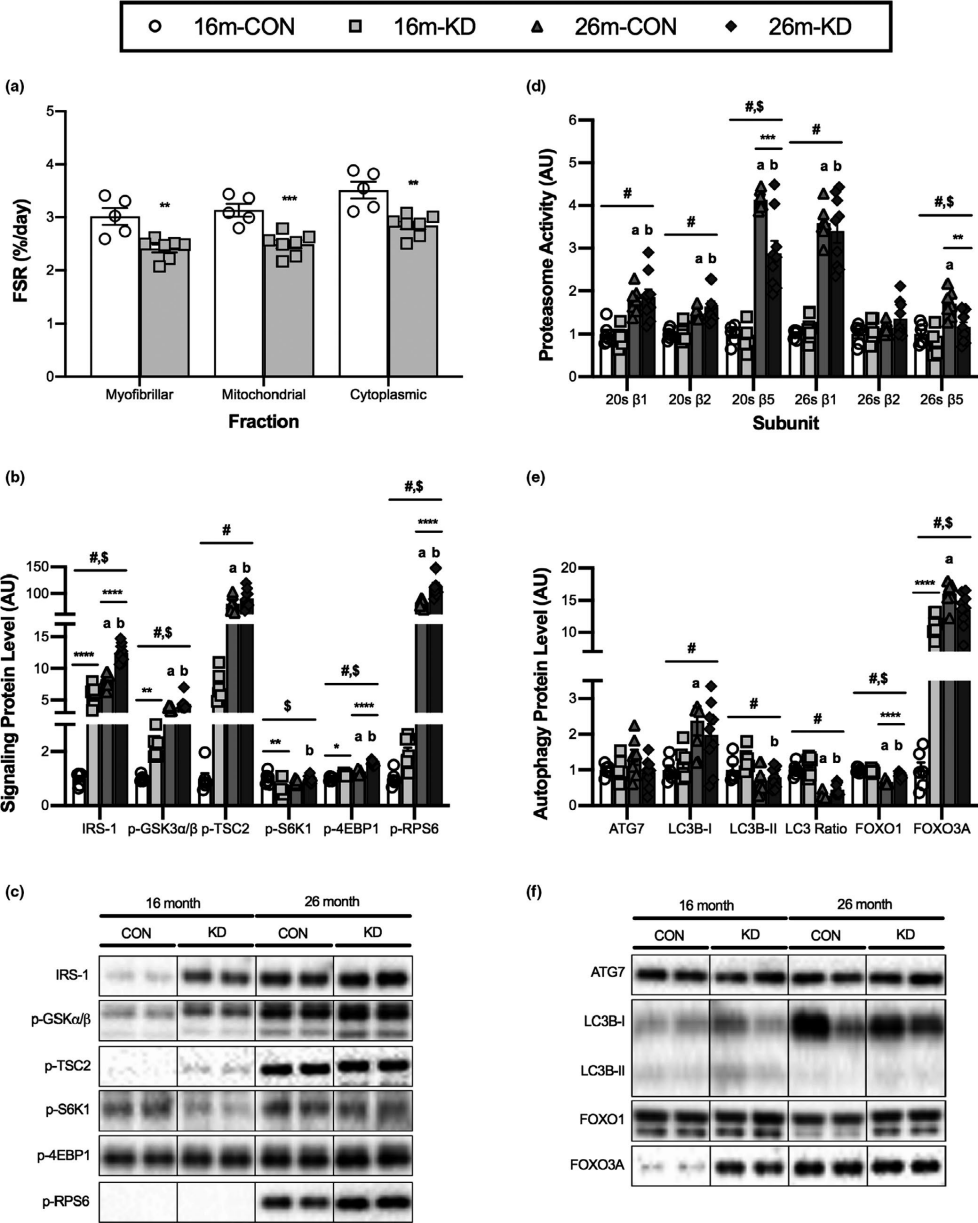
Effect of diet and aging on skeletal muscle proteostasis. (a) Fractional synthesis rate (FSR) of the myofibrillar, cytoplasmic, and mitochondrial fractions from the GTN of 16‐month‐old mice on a control (CON) or ketogenic diet (KD). Values are expressed as means ± SEM. (*p < 0.05; **p < 0.01; ***p < 0.001; ****p < 0.0001) comparing CON and KD (b, c) Protein levels and representative western blot images of proteins associated with protein synthesis from the GTN of 16‐ and 26‐month‐old mice on a CON or KD. (d) ATP‐independent (20S) and ATP‐dependent (26S) proteasomal subunit (β1, β2, and β5) activities from the quadricep of 16‐ and 26‐month‐old mice on a CON or KD. (e, f) Protein levels and representative western blot images of proteins associated with autophagy from the GTN of 16‐ and 26‐month‐old mice on a CON or KD. For complete western blot images, refer to Appendix S1 (Figure S4). Values are expressed as means ± SEM. *n* = 6 (16‐month CON), *n* = 7 (16‐month KD and 26‐month CON), and *n* = 9 (26‐month KD). (#) main effect of age. ($) main effect of diet. (*p < 0.05; **p < 0.01; ***p < 0.001; ****p < 0.0001) comparing CON and KD at 16 and 26 months. (a) *p* < 0.05 comparing CON at 16 and 26 months. (b) *p* < 0.05 comparing KD at 16 and 26 months

To determine whether changes in protein synthesis and/or protein degradation could explain the improved maintenance of muscle mass with aging seen on the KD, measurements of muscle protein synthesis and markers of proteolysis were made in the GTN muscle. Using deuterium oxide, protein synthesis was measured over the final two weeks of the diet and showed a significant decrease in protein synthesis rates in the myofibrillar, mitochondrial, and cytoplasmic fractions of 16‐month‐old mice on a KD diet compared with CON (Figure 5a). As the mTORC1 signaling pathway serves as a regulator of cellular growth, we investigated key upstream and downstream signaling proteins (Figure 5b,c). The upstream regulator of mTORC1 IRS‐1 was significantly elevated at 26 months for both CON and KD when compared to 16‐month levels. Similarly, Akt activity (determined through GSK3α/β phosphorylation) was elevated at 26 months for both CON and KD. The level of IRS‐1 was significantly higher for the KD group when compared to CON at 16 and 26 months, while p‐GSK3α/β was only significantly higher in the KD group when compared to CON at 16 months. Diet had no effect on TSC2 (p‐TSC2) phosphorylation at the AMPK (Ser1345) site; however, there was a significant increase in TSC‐2 phosphorylation between 16 and 26 months for both CON and KD. The phosphorylation of downstream targets of mTORC1, p‐S6 K1, and p‐4EBP1 was lower and higher, respectively, at 16 months of age in the KD group compared to CON. With aging, p‐4EBP1 and p‐rpS6 increased significantly between 16 and 26 months for both diets; however, only the KD significantly increased p‐S6 K1 between 16 and 26 months. At 26 months, p‐4EBP1 and p‐rpS6 levels were significantly higher in the KD group when compared to CON.

Protein degradation was estimated by measuring the ATP‐independent (20S) and ATP‐dependent (26S) activity of the three proteolytic subunits (β1, β2, and β5) of the proteasome in the QUAD muscle (Figure 5d). There was a main effect of age on 20S β1, β2, β5 and 26S β1, β5 activity, in that proteasome activity increased between 16 and 26 months of age. We observed a main effect of diet for β5 activity, with the activity of these subunits being lower in the KD group. The activity of 20S β5 increases for both CON and KD between 16 and 26 months; however, mice on the CON diet showed significantly higher activity at 26 months when compared with KD. At 26 months, the activity of the 26 β5 subunit was significantly elevated in CON when compared to KD, this level was also significantly higher when compared to CON at 16 months.

As the autophagy degradation system is another system that regulates protein breakdown, we set out to determine the effect of diet and age on the levels of autophagy‐related markers (Figure 5e,f). There was no effect of diet or age on the levels of ATG7. There was a main effect of age on LC3B protein levels, in that LC3B‐I was higher and LC3B‐II and the LC3B‐II to LC3B‐I ratio were lower with age (suggesting decreased autophagy with age). There was no effect of diet on LC3B levels. From 16 to 26 months, there was a significant increase in LC3B‐I levels for CON, whereas there was a decrease in the level of LC3B‐II for KD. When analyzing the LC3B‐II to LC3B‐I ratio, we observed a significant decrease between 16 and 26 months for both diets. There was a main effect of age and diet on FOXO1 and FOXO3A levels, with age decreasing FOXO1 and increasing FOXO3A, while both FOXO1 and FOXO3A were increased with the KD. There was a significant decline in FOXO1 levels between 16 and 26 months of age for both diets; however, at 26 months, FOXO1 levels were significantly lower for CON when compared to KD. FOXO3A levels were significantly higher for KD when compared to CON at 16 months and remained at this level at 26 months. For the CON diet, the level of FOXO3A significantly increased from 16 to 26 months.

## 3 DISCUSSION

The objective of this study was to begin identifying mechanisms whereby a KD results in the preservation of skeletal muscle mass and function with age in mice. Our data, using either 4 or 14 months on an 11.2 kcal/day standard control or KD, suggest that a KD results in preservation of skeletal muscle mass concomitant with a decrease in type IIb and increase in type IIa fiber area. At the molecular level, the KD increased markers of NMJ turnover, mitochondrial biogenesis, oxidative metabolism, and oxidative stress response, while decreasing ER stress, protein synthesis, and proteasome activity. Together, these data suggest that a KD results in a healthier cellular environment, decreasing the need for protein turnover and ER stress. However, what is most clear from our data is that no single molecular target drives the improvement in muscle function on this diet.

### 3.1 Skeletal muscle weight, fiber size and type

Skeletal muscle mass was better maintained in 26‐month‐old mice fed a KD compared to CON diet. Together with the increase in grip strength and wire hang performance previously reported in this cohort of animals (Roberts et al., 2018), this indicates that muscle mass and function are improved on a long‐term KD. The fact that the grip strength and wire hang used previously focus on forearm muscle and the improved muscle mass observed in the current study focused on the hindlimb muscles, suggests that a KD influences muscle throughout the body. The improved muscle mass observed in the current work is similar to a recent study showing ketone diesters mitigate cachexic muscle loss (Koutnik et al., 2020). Furthermore, this maintenance of muscle mass was biased within more oxidative (type IIa) fibers at the expense of type IIb fiber area. This shift in fiber type area is important because in humans the loss of IIa fiber CSA could explain the loss of muscle size and strength with age (Nilwik et al., 2013). It is unclear whether preferential sparing of oxidative fibers was due to a loss in glycolytic fibers, a fiber‐type shift from glycolytic to oxidative fiber types as a result of an increased reliance on fat as a fuel, or enhanced reinnervation of IIb fibers by type I, IIa, or IIx motor neurons. Based on our data, we hypothesize that there was an increase in axonal sprouting and fiber grouping and together with the shift in fuel utilization required to sustain a KD (i.e., increased aerobic respiration and beta‐oxidation), this led to preservation of oxidative fibers over glycolytic ones (Lin et al., 2014; Plomgaard et al., 2006).

### 3.2 Neuromuscular junction remodeling

One theory for the progressive loss of muscle fiber CSA and number is based on progressive cycles of denervation and reinnervation that muscle fibers undergo during aging (Rudolf et al., 2014). This theory posits a neurogenic basis for fiber loss resulting from neuromuscular fragility and progressive reinnervation by axonal sprouting from an adjacent motor neuron. In fact, individuals where axonal sprouting was increased, as measured through expanded motor unit size, were less likely to be sarcopenic than those whose motor unit size remained small (Piasecki et al., 2018). In support of the theory that axonal sprouting improves muscle mass and function with age, masters athletes showed greater reinnervation capacity than an age‐matched frail elderly group (Sonjak et al., 2019). Sonjak and colleagues concluded that the difference in age‐related muscle function was related to the robustness of the reinnervation response. In the current study, we observed more fiber clustering (type IIa) in animals on a KD, which is typically the result of increased axonal sprouting. The increase in axonal sprouting was seen even though the expression of genes associated with denervation was augmented by the KD. These results suggest that the preservation of muscle mass and function observed with a KD is not the result of a reduction in the rate of fiber denervation but that reinnervation through axonal sprouting of IIa nerves may contribute to improved muscle function.

### 3.3 Mitochondrial biogenesis and oxidative metabolism

Skeletal muscle aging and the onset of sarcopenia has been shown to be driven metabolically through disruptions in mitochondrial function (Coen et al., 2018). It is important to note that markers of mitochondrial mass (i.e., levels of OXPHOS proteins) and function (i.e., citrate synthase activity) increased between 16 and 26 months in the current study. Even though this went contrary to our hypothesis, an increase in mitochondrial protein synthesis with age has been reported previously (Miller et al., 2019). Beyond the effect of age, citrate synthase activity improved even more as a result of the forced metabolic shift of a short‐term (4 months: 16‐month‐old) or long‐term (14 months: 26‐month‐old) KD. The KD led to multiple intracellular signals converging to drive markers of mitochondrial biogenesis in both age groups. A short‐term KD induced a robust increase in *Tfam* mRNA and an increase in SIRT1, SIRT3, and PGC‐1α protein, suggestive of mitochondrial biogenesis. By contrast, direct measurement of mitochondrial protein synthesis during the last two weeks of a four‐month KD showed that mitochondrial protein synthesis decreased. Even with a decrease in mitochondrial protein synthesis, animals on a KD had higher citrate synthase activity, indicating either that a KD either increased mitochondrial biogenesis (and protein synthesis) at an earlier time point or that a KD increased one measure of mitochondrial activity independent of mass. The fact that OXPHOS protein levels were not different between diets at 16 months argues that a short‐term KD increases mitochondrial activity. A long‐term KD also showed molecular signals consistent with mitochondrial biogenesis including increased *Ppargc1a* expression, the angiogenic factor *Esrra*, and the mitochondrial regulators *Tfam and Tfb1 m*. In the older mice on a KD, the increase in markers of mitochondrial biogenesis was matched with greater citrate synthase activity (a proxy for mitochondrial function) and higher levels of mitochondrial oxidative phosphorylation machinery (complexes I, III, IV, and V). Together, these data suggest that a KD may increase mitochondrial activity in younger animals (shorter time on the diet) and mitochondrial mass and function in older animals. This improved mitochondrial function may contribute to the improved longevity on a KD (Roberts et al., 2018). However, the direct contribution of greater mitochondrial function to muscle mass and strength with age remains to be determined.

### 3.4 Cellular stress response

One of the most striking findings of the current work was that the age‐associated increase in ER stress was completely attenuated by a KD. On the control diet, aging resulted in an increase in BiP, IRE1α, and PDI. This is consistent with our previous work in aging muscle (Baehr et al., 2016). By contrast, each of these factors were lower in mice on a long‐term KD. Whereas the chaperones (BiP and PDI) of the adaptive ER stress response were lower on a KD, the protein synthesis regulator eIF2α was significantly more phosphorylated in response to both a short‐term and long‐term KD. In fact, the phosphorylation of eIF2α appears more predictive of the decrease in protein synthesis on a KD than other molecular regulators (e.g., mTORC1; see below). These data suggest that a KD decreases unfolded proteins resulting in a healthy cellular milieu.

Maintaining redox status is also particularly important for preserving cellular homeostasis. Increased production of reactive oxygen species (ROS) and a reduction of antioxidant scavengers with aging are thought to contribute to dysfunction and exercise intolerance through genomic and proteomic oxidation (Deepa et al., 2019; Sakellariou et al., 2018). In this study, we see a decrease in SOD2 with age that is prevented by long‐term KD. Similarly, catalase tends to decrease with age and increases with both short‐ and long‐term KD. An increase in antioxidants with a KD is consistent with the increase in mitochondrial activity, and presumably ROS production, with the increased reliance on fat as a fuel. Depletion of SOD2 and catalase is sufficient to induce muscle dysfunction and inhibit mitochondrial function in mice (Lee et al., 2010; Lustgarten et al., 2011). Further, the loss of catalase has been implicated in muscle atrophy (Sullivan‐Gunn & Lewandowski, 2013). Therefore, the ability of a long‐term KD to increase ROS scavenging capacity, possibly in response to the increased electron transport necessary for fat oxidation, may protect muscle from oxidative stress during aging. However, it is important to note that Picard and colleagues have shown that SOD and catalase activity increase the most in muscles that show the greatest decrease in muscle mass with age (Picard et al., 2011), suggesting that a simple relationship between increased ROS scavenging and maintained muscle mass does not exist.

Age‐associated changes to the immune system and low‐grade chronic inflammation have been suggested to contribute to skeletal muscle loss with aging (Nelke et al., 2019). In skeletal muscle, the NFκB pathway is a key transcriptional regulator of pro‐inflammatory factors, such as Il‐1β, which in turn is activated by the NOD‐like receptor family pyrin domain containing 3 (NLRP3) inflammasome (Liu et al., 2017). With aging, the level of NFκB subunits (p50 and p65), Il‐1β transcription and the activity of the NLRP3 inflammasome have previously been shown to increase in older skeletal muscle (McBride et al., 2017; Peake et al., 2010). We have previously shown that KD had no effect on the levels of plasma inflammatory markers (TNFα, CXCL1, and IL‐6) when compared to an isocaloric control diet (Roberts et al., 2018). In the present study, we similarly show that aging had no effect on NFκB signaling (p50 and p65) or IL‐1β expression in skeletal muscle and this was not affected by diet. The fact that NFκB signaling and IL‐1β mRNA expression were not elevated with age suggests that keeping the animals weight‐neutral throughout the study may have prevented the typical rise in inflammation seen in aging animals. Consistent with this hypothesis, CR reduces inflammation in muscle (Lopez‐Lluch & Navas, 2016). Therefore, a KD may reduce inflammation in conditions where metabolic syndrome is present and inflammation is elevated, but this does not appear to be an important mechanism during healthy aging.

### 3.5 Proteostasis

Muscle protein synthesis rates were determined using deuterated water in mice over the last two weeks of a 4‐month KD. These data indicated that myofibrillar, mitochondrial, and cytoplasmic fractional synthesis rates were all decreased by a KD. We were unable to collect fractional synthesis rates for mice on the 16‐month KD or determine DNA synthesis rate at any time point. The decrease in fractional synthesis observed at 16 months matches that of other pro‐longevity models that inhibit growth signaling pathways to downregulate protein synthesis (Drake et al., 2013). Interestingly though, our data diverge from these growth‐restriction, pro‐longevity models as the KD increased IRS‐1 levels, which might imply greater potential for activation of mTORC1, while attenuating protein synthesis. As stated above, the decrease in protein synthesis on a KD best fits with the increase in eIF2α phosphorylation in response to both short‐ and long‐term KD. eIF2 phosphorylation inhibits protein synthesis by blocking the formation of the 43S preinitiation complex. Formation of the preinitiation complex requires guanine nucleotide exchange on eIF2. This process is facilitated by the guanine nucleotide exchange factor eIF2B (Siekierka et al., 1982) and is regulated in times of decreased nutritional supply and diabetes (Proud & Denton, 1997). Upon phosphorylation of the alpha subunit, eIF2 shifts from a substrate to an inhibitor of eIF2B (Kimball, 1999). Binding of phosphorylated eIF2α to eIF2B sequesters eIF2B in inactive complexes. Since eIF2 outnumbers eIF2B by 10 to 1, this quickly inhibits GTP exchange and therefore the initiation of protein synthesis (Bogorad et al., 2018). A decrease in translation initiation may slow protein synthesis and increase the fidelity of translation, preventing protein misfolding and decreasing ER stress. Further, since translational fidelity is inversely related to longevity (Ke et al., 2017), a decrease in translational initiation could contribute to the increase in longevity observed on a KD (Roberts et al., 2018).

In addition to reduced protein synthesis, proteasomal degradation was marginally attenuated by a long‐term KD. Mean proteasomal degradation was greater at 26 than 16 months of age. The increase in activity with age is contrary to what is seen between 6 and 24 months of age in rats, where proteasome activity decreases in skeletal muscle (Hwee et al., 2014). Similarly, Selsby and colleagues and Radak et al found that proteasome activity decreased between 10 and 28–30 months in Fisher 344 rats (Radak et al., 2002; Selsby et al., 2005). These findings are in contrast to what has been observed in Fischer 344 X Brown Norway F1‐hybrid animals where proteosome activity increases between 8 and 30–40 months (Hepple et al., 2008). These discrepancies may be the result of genetic differences between the strains or that proteasome activity starts high in younger animals where muscle growth rate is higher (Baehr et al., 2014), decreases to a nadir between 16 and 18 months and then begins to increase from there. The increase we observed from 16 to 26 months tended to be lower with the KD, specifically for 20 s β5 and 26 β5 activity. This is similar to what has been observed with caloric restriction (Hepple et al., 2008). For autophagy, aging appeared to have a more dramatic effect than the KD. The LC3B II:I ratio decreased dramatically between 16 and 26 months, suggestive of a decrease in autophagy with aging. This is consistent with previous work on autophagy in aging mouse muscle (Carnio et al., 2014). Activators of autophagy have been shown to improve muscle function with age; however, a long‐term KD did not affect autophagy. The only atrophy‐associated proteins that were responsive to the KD were FOXO1 and FOXO3a. FOXO1 was significantly higher in KD than CON mice at 26 months old, whereas FOXO3a was significantly elevated by short‐term KD, nearly mimicking the heightened FOXO3a levels at 26 months old in both CON and KD mice compared to young controls. Overall, the marginal decrease in protein degradation is probably the result of a healthier cellular environment (less oxidative and ER stress) decreasing the need for turnover. This hypothesis is further supported by the reduced protein synthesis rates observed in the KD mice. Given the marginal effect on degradation, it is unlikely that degradation is responsible for the increase in muscle mass and function on a KD.

## 4 CONCLUSION

**FIGURE 6:**
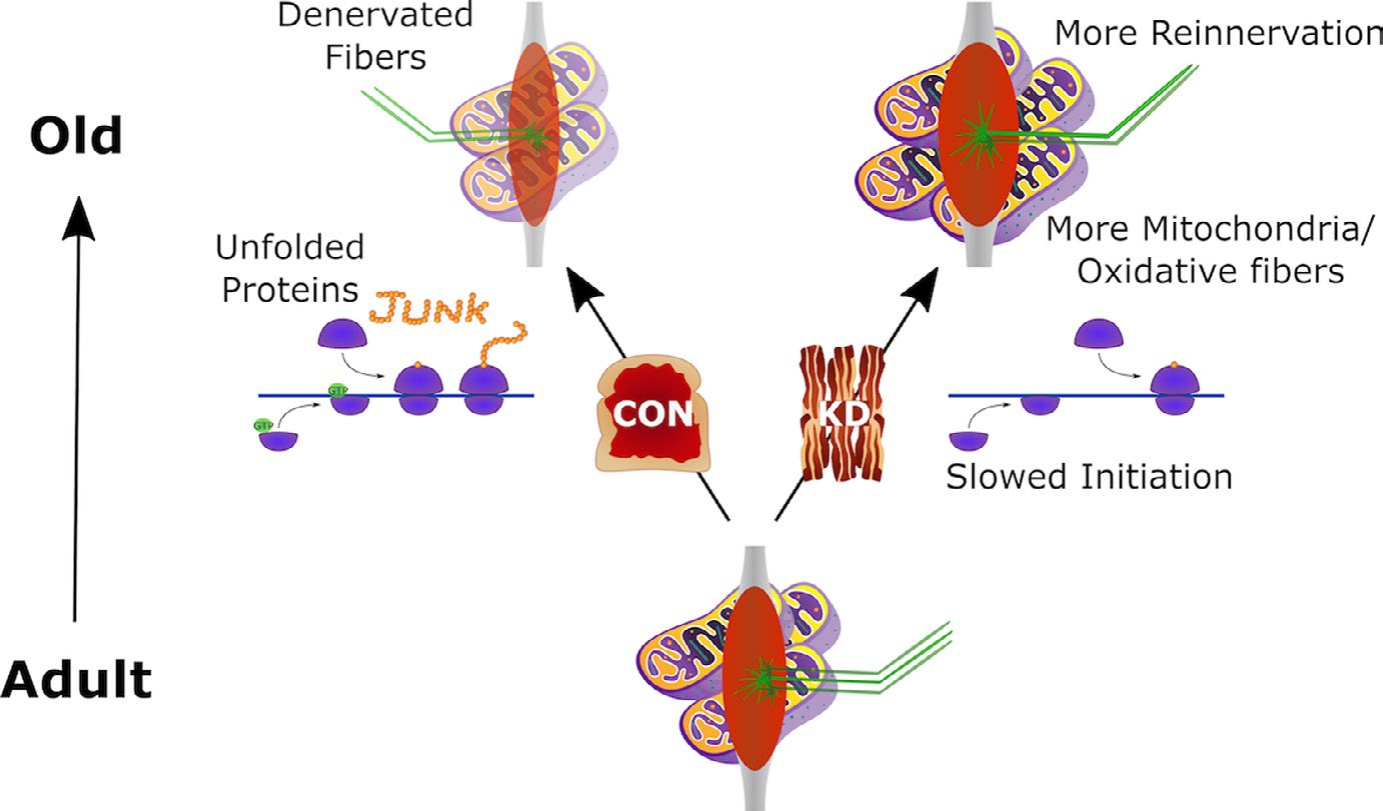
Potential mechanism of the effect of the ketogenic diet (KD) on muscle preservation with aging. As muscle progresses from adult to old, individuals on a standard control (18% PRO, 65% CHO, and 17% FAT) diet show less reinnervation and more unfolded proteins. By contrast, individuals on a long‐term KD (10% PRO, <1% CHO, and 89% FAT) showed more mitochondria, greater reinnervation, more oxidative muscle fibers, and decreased translation initiation

Overall, this study shows the effectiveness of a long‐term ketogenic diet in mitigating sarcopenia. As summarized in Figure 6, a ketogenic diet increased mitochondrial and antioxidant proteins possibly leading to the preferential preservation of oxidative muscle fibers. The increase in fiber grouping in old animals on a ketogenic diet suggests that axonal sprouting and reinnervation may have increased helping to maintain muscle mass. In both young and old animals, we observed an increase in the phosphorylation of eIF2α that would slow translation initiation and may result in improved translational fidelity and a healthier cellular environment wherein mice on a KD would require less protein turnover, measured as reduced protein synthesis, proteasomal degradation, and ER stress. Together, these shifts would allow mice to maintain a greater muscle mass as they age. However, it is also plausible that the benefits observed in muscle mass and function may be secondary to changes in other tissues, since a long‐term KD is an organismal rather than tissue‐level intervention.

## 5 EXPERIMENTAL PROCEDURES

### 5.1 Animals

Animals utilized for this study were in part from a larger study that specifically examined the effect of dietary interventions on life span and health span (Roberts et al., 2018). Adult (11 months) C57BL/6 mice were obtained from the NIA Aged Rodent Colony and housed in individually housed in a HEPA filtered room maintained on a 12‐hr light–dark cycle. The mice were fed a chow diet (LM485, Envigo, Madison, WI) ad libitum and were allowed to acclimatize for 1 month. All animal protocols were approved by the UC Davis Institutional Animal Care and Use Committee and were in accordance with the NIH guidelines for the Care and Use of Laboratory Animals.

### 5.2 Dietary intervention

At 12 months of age, mice were randomly placed on an isocaloric (11.2 kcal/day) control (CON) or ketogenic diet (KD). The CON diet contained (% of total kcal) 18% protein, 65% carbohydrate, and 17% fat. The KD contained 10% protein, <1% carbohydrate, and 89% fat. Mice were maintained on these diets until sacrificed at 16 (CON: *n* = 6, KD: *n* = 7) and 26 months (CON: *n* = 7, KD: *n* = 9) of age. Full details of the diets have previously been provided (Roberts et al., 2018).

### 5.3 Tissue collection

Following completion of the dietary intervention, all mice were fasted overnight prior to tissue collection the following day. Mice were weighed and then anesthetized with 2% inhaled isoflurane for tissue removal, prior to being sacrificed. The quadricep (QUAD), gastrocnemius (GTN), plantaris (PLN), soleus (SOL), tibialis anterior (TA), and extensor digitorum longus (EDL) muscles were removed, weighed, and then frozen in liquid nitrogen for biochemical analyses or were pinned at resting length and flash frozen in liquid nitrogen‐cooled isopentane for histological analysis.

### 5.4 Immunohistochemistry

Please refer to Appendix S1 for more details.

### 5.5 Determination of fractional protein synthetic rate

Please refer to Appendix S1 for more details.

### 5.6 Proteasome activity

Please refer to Appendix S1 for more details.

### 5.7 Citrate synthase activity

Please refer to Appendix S1 for more details.

### 5.8 RNA analysis

Please refer to Appendix S1 for more details.

### 5.9 Western blotting

Please refer to Appendix S1 for more details.

### 5.10 Statistical analysis

Please refer to Appendix S1 for more details.

## Conflict of Interest

Authors declare no competing interests.

## Author contributions

M.N.R., J.A.L‐D., and J.J.R. contributed to conceptualization of the study. M.A.W., N.W.A, and K.B. developed the design and methodology for the muscle‐specific aspects of the study. M.A.W., N.W.A., G.R.M., A.G.M., L.M.B., D.C.H., K.L.H., M.N.R., J.A.L.‐D., B.F.M., and J.J.R. contributed to data collection. M.A.W, N.W.A., and K.B carried out the formal analysis. M.A.W, N.W.A, and K.B wrote the manuscript. All authors approved the final manuscript.

## Supporting information

Supplementary Material

## Data Availability

All data are available in the manuscript or the supplementary materials. Correspondence and requests for information should be addressed to corresponding author M.A.W.
