# Supplementary material for: The ketogenic diet preserves skeletal muscle with aging in mice
Source: Aging Cell. 2021 Mar 6;20(4):e13322. doi: 10.1111/acel.13322 (PMC8045940; doi:10.1111/acel.13322)
Supplement: Supplementary file 1 — Supplementary Material [file ACEL-20-e13322-s001.docx]

**Appendix S1**

**Supplementary Experimental Procedures**

**Immunohistochemistry**

Serial cross sections (10 μm) were cut from the GTN using a Leica CM 3050S cryostat. To determine fiber-type-specific cross-sectional area (CSA), muscle sections were fixed in cold acetone at −20°C for 5 min. Between each step, sections underwent three 5-minute washes in phosphate-buffered saline with 0.1% Tween-20. Sections were blocked using the M.O.M. (Mouse on Mouse) Immunodetection Kit (#BMK-2202, Vector) for 1 hour at room temperature, then incubated in primary antibodies overnight at 4°C. A polyclonal laminin antibody (1:500, #L9393, Sigma-Aldrich) was diluted 1:500 in blocking buffer and included for the determination of CSA. Fiber type specific primary antibodies (BA-F8 (slow type Mm, IgG2B); SC-71 (myosin heavy chain 2A, Mm, IgG1); BF-F3 (myosin heavy chain 2B, Mm, immunoglobulin M)) were diluted 1:250 in blocking buffer and were was deposited to the Developmental Studies Hybridoma Bank (Iowa City, IA) by Stefano Schiaffino. Sections were subsequently incubated in secondary antibodies for 30 min at RT. Goat-anti-rabbit AlexaFluor 647 (Life Technologies) was used to detect laminin, while fluorescently conjugated goat-anti-mouse immunoglobulin-specific secondary antibodies (Alexa Fluor 350, 488, and 555, Life Technologies) were used for simultaneous detection of multiple mouse primary antibodies. Sections were cover-slipped using ProLong Gold Antifade reagent (#P36930, Life Technologies) and imaged using a Zeiss Axio Imager.M1 fluorescent microscope using the EC Plan-Neofluar ×10 objective. Representative images were taken from ten regions of a single section per animal and fiber CSA and fiber type was analyzed using Axiovision software. Fiber clustering of type IIa fibers was measured manually and was defined by any type IIa that was adjacent to two or more other type IIa fibers.

**Determination of fractional protein synthetic rate**

Newly synthesized proteins were labeled with deuterium oxide (D2O) using previously published procedures (Drake et al., 2015; Drake et al., 2013; Reid et al., 2020). Two weeks prior to sacrifice, the 16-month cohort received an intraperitoneal (i.p) bolus injection of 99% D2O, followed by 8% D2O enriched drinking water. Approximately 20-25 mg of skeletal muscle was pulverized using liquid nitrogen-cooled mortar and pestle. In brief, tissue was fractionated into subcellular compartments by differential centrifugation as described previously (Drake et al., 2015; Drake et al., 2013; Reid et al., 2020) to obtain myofibrillar, mitochondrial and cytosolic protein fractions. In addition, plasma was prepared using distillation for the determination of precursor pool enrichment. Protein fractions were derivatized for analysis of deuterium-enrichment by gas chromatography-mass spectrometry (Agilent 7890A GC and 5975C MS). Deuterium enrichment of protein was used to calculate protein fraction new using a MIDA correction (Hellerstein & Neese, 1999) for the equilibration of the precursor enrichment with the body water pool (Drake et al., 2015; Drake et al., 2013; Reid et al., 2020).

**Proteasome activity**

Analysis of 20S and 26S proteasome activities were performed as previously described (Baehr et al., 2016; Gomes et al., 2006). Proteasome activities were determined by adding substrates at 100 µM: Z-Leu-Leu-Glu-MCA (#3179-v, Peptide Institute), Boc-Leu-Ser-Thr-Arg-AMC (#I-1940, Bachem), or succinyl-Leu-Leu-Val-Tyr-7-AMC (#I-1395, Bachem), for β1- (caspase-like), β2- (trypsin-like), or β5-subunits (chymotrypsin-like), respectively. Each assay was conducted in the absence and presence of proteasome inhibitor bortezomib (#2204, Cell Signaling) at a final concentration of 10 mM (β1 and β2) or 2 mM (β5). The activity of the 20S and 26S proteasome was measured by calculating the difference between fluorescence units recorded with or without the inhibitor in the reaction medium. Fluorescence was measured using a Fluoroskan Ascent fluorometer (Thermo Electron, excitation wavelength, 390 nm; emission wavelength, 460 nm) at 15-min intervals for 2 hours.

**Citrate Synthase Activity**

Citrate synthase (CS) activity was measured as previously described (Wadley & McConell, 2007). Muscle homogenates from the GTN western blot preparation were used to measure CS activity spectrophotometrically by following the increase in 5,5′-dithiobis-2-nitrobenzoate (DTNB) at 412 nm. CS activity was expressed in μmol min−1 g−1 of total protein.

**RNA Analysis**

Aliquots of frozen GTN were weighed to calculate amount of RNA per milligram of wet muscle tissue. Total RNA was extracted from ~20 μg of tissue using Tri Reagent® Solution (#AM9738, Ambion Inc.) according to manufacturer’s instructions. RNA concentrations were determined using the Epoch Microplate Spectrophotometer (BioTek Instruments Inc.). cDNA was synthesized using the High-Capacity cDNA Reverse Transcription Kit (#4368814, Life Technologies) from 1 μg of total RNA according to the manufacturer’s instructions. Gene expression was analyzed by quantitative PCR (qPCR) using SYBR Green Supermix (#172–5121, Bio-Rad Laboratories) on an 7900HT Fast Real-Time PCR System (Thermo Scientific). Primer sequences are available on request. Gene expression was calculated using the delta threshold cycle method and then normalized to the amount of RNA per milligram of tissue used (Heinemeier et al., 2007). This method of normalization was selected due to no reference gene remaining constant with age and diet.

**Western Blotting**

Total protein was homogenized from frozen GTN muscles in sucrose lysis buffer (50 mM Tris, pH 7.5, 250 mM sucrose, 1 mM EDTA, 1 mM EGTA, 1% Triton X-100, 50 mM NaF) with added protease and phosphatase inhibitors (#88668, Pierce). Cytosolic and nuclear protein fractions were extracted from frozen QUAD muscles by first homogenizing in STM buffer (1M sucrose, 1M Tris, pH 7.4, 1M MgCl2, water, and protease inhibitors). The homogenate was centrifuged at 800 x g for 15 minutes. Supernatants were transferred to a new tube and centrifuged for 10 minutes at 11,000 x g. The resulting supernatant was then transferred to a new tube and labeled Cytosolic Fraction. The pellet from the initial centrifugation was resuspended in STM buffer and vortexed for 15 sec. The resuspended pellet was centrifuged at 800 x g for 15 minutes. The supernatant was discarded, and the pellet was resuspended in STM buffer, followed by two cycles of centrifugation as described above. Following the final centrifugation, the pellet was resuspended in NET Buffer (1 M HEPES, 1M MgCl2, 3M NaCl, 100mM EDTA, Glycerol, 1% Triton-X 100, water, and protease inhibitors). The resulting mixture was sonicated for 10 seconds and then centrifuged at 11,000 x g for 15 minutes. The supernatant was transferred to a new tube and labeled Nuclear Fraction.

Concentration of the total, cytosolic and nuclear proteins was determined using the DC protein assay (Bio-Rad) according to manufacturer’s instructions. Twenty micrograms of protein were then subjected to SDS-PAGE on 4–20% Criterion TGX stain-free gels (#5678095, Bio-Rad) and transferred to polyvinylidene difluoride (PVDF) membrane (#IPVH00010, EMD Millipore). Membranes were blocked in 1% fish skin gelatin in Tris-buffered saline with 0.1% Tween-20 for 1 hour and then probed with primary antibody at 1:1000 overnight at 4°C. The next day, membranes were washed and incubated with horseradish peroxidase-conjugated secondary antibodies at 1:10,000 for one hour at RT. Immobilon Western Chemiluminescent horseradish peroxidase substrate (Millipore) was then applied to the membranes for protein band visualization by chemiluminescence. Image acquisition and band quantification was performed using the ChemiDoc™ MP System and Image Lab 5.0 software (Bio-Rad). Total protein staining of the membrane (via Ponceau) was used as the normalization control for all blots. Primary antibodies used for immunoblotting were from Cell Signaling Technology (p-4EBP1^Thr37/46^, #9459; p-AMPK^Thr172^, #2535; AMPK, #2532; ATG7, #2631; BiP, #3177; Catalase, #14097; CHOP, #2865; p-eIF2α^Ser51^, #9721; FOXO1, #2880; FOXO3A, #2497; p-GSK3α/β^Ser21/9^, #8566; IRE1α, #3294; IRS-1, #2382; LC3B, #2775; p-p38 MAPK^Thr180/Tyr182^, #9211; p38 MAPK, #9212; NFκB p65, #4764; NFκB p105/p50, #13586; PDI, #3501; p-p70 S6 Kinase^Thr389^, #9205; p-S6 Ribosomal Protein ^Ser240/244^, #2215; SIRT1, #9475; SIRT3, #5490; p-TSC2^Ser1387^, #5584), Abcam (PGC1α, #ab54481; Total OXPHOS Antibody Cocktail, #ab110413) and Santa Cruz Biotechnology (SOD2, #sc-137254).

**Statistical Analysis**

Results are presented as mean ± SEM. Differences between diet at 16 months for fractional synthetic rate (FSR) were analyzed by Student’s t test. Differences between diet groups and ages were evaluated using a two-way ANOVA using GraphPad Prism software (GraphPad Software, Inc., La Jolla, CA). Tukey’s post hoc analysis was used to determine the differences when interactions existed. Statistical significance was set at p <0.05.

**Supplementary Figures**


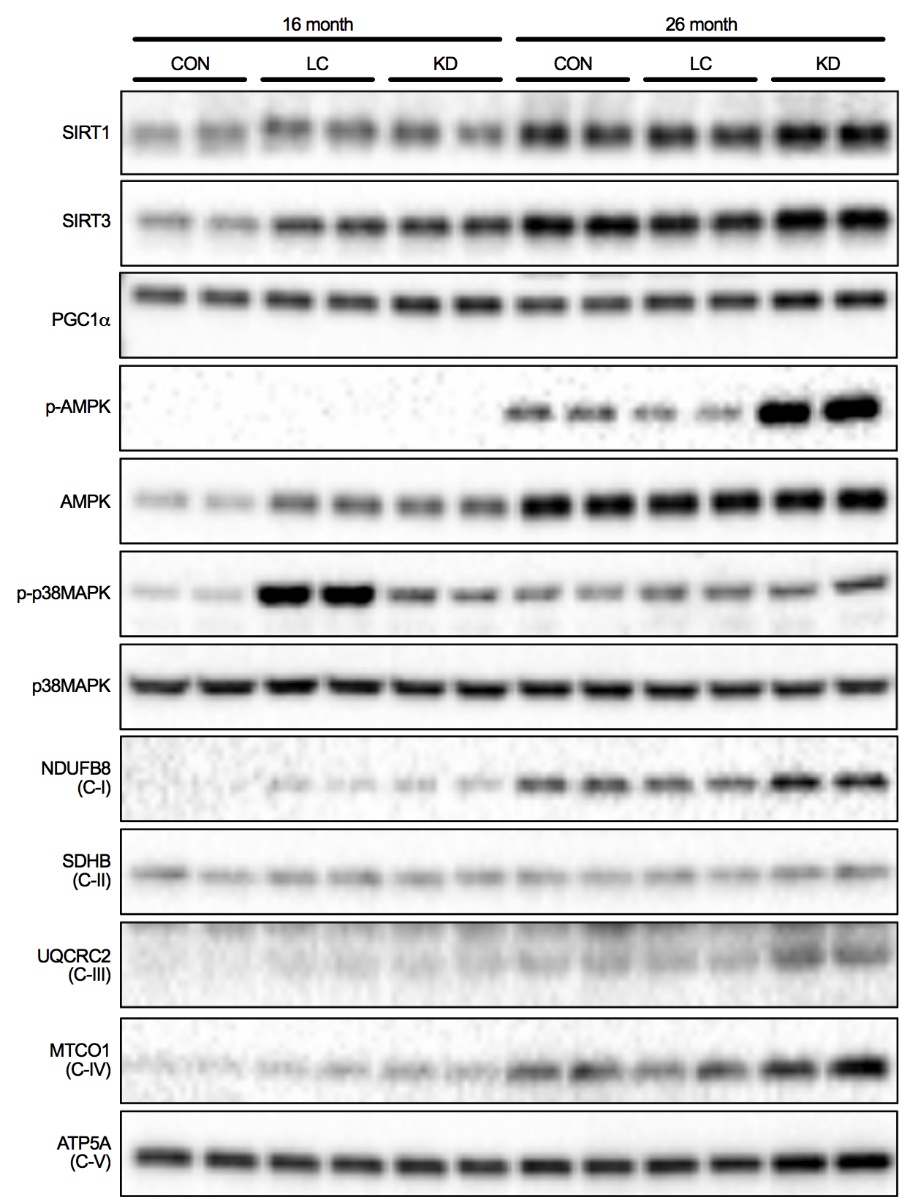


**Figure S1.** Complete western blot images of representative images illustrated in Figure 3D & F. Bands are from total proteins extracted from the GTN of 16 and 26 month mice on a control (CON), low-carbohydrate (LC) and ketogenic diet (KD). Due to the exclusion of the LC group from the study, these images were edited to remove the LC group from the representative images.


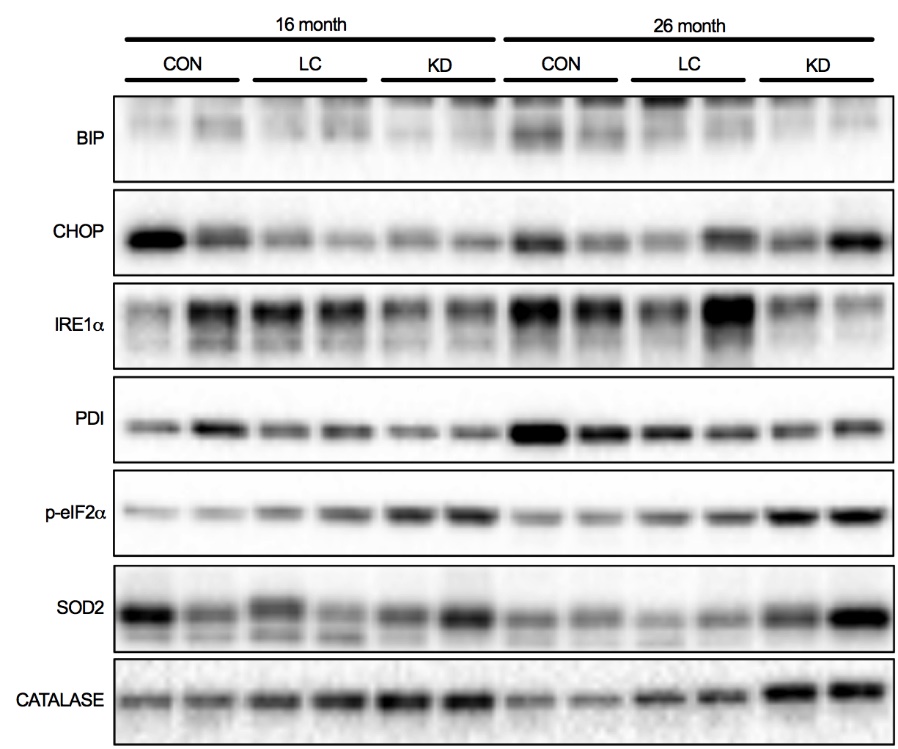


**Figure S2.** Complete western blot images of representative images illustrated in Figure 4B & D. Bands are from proteins extracted from the GTN of 16 and 26 month mice on a control (CON), low-carbohydrate (LC) and ketogenic diet (KD). Due to the exclusion of the LC group from the study, these images were edited to remove the LC group from the representative images.


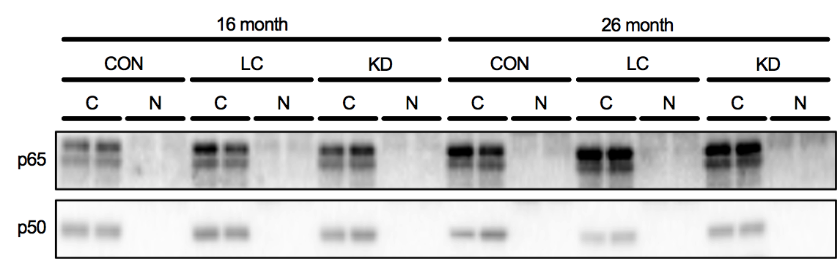


**Figure S3.** Complete western blot images of representative images illustrated in Figure 4F. Bands are from cytoplasmic (C) and nuclear (N) protein fractions extracted from the QUAD of 16 and 26 month mice on a control (CON), low-carbohydrate (LC) and ketogenic diet (KD). Due to the exclusion of the LC group from the study, these images were edited to remove the LC group from the representative images.


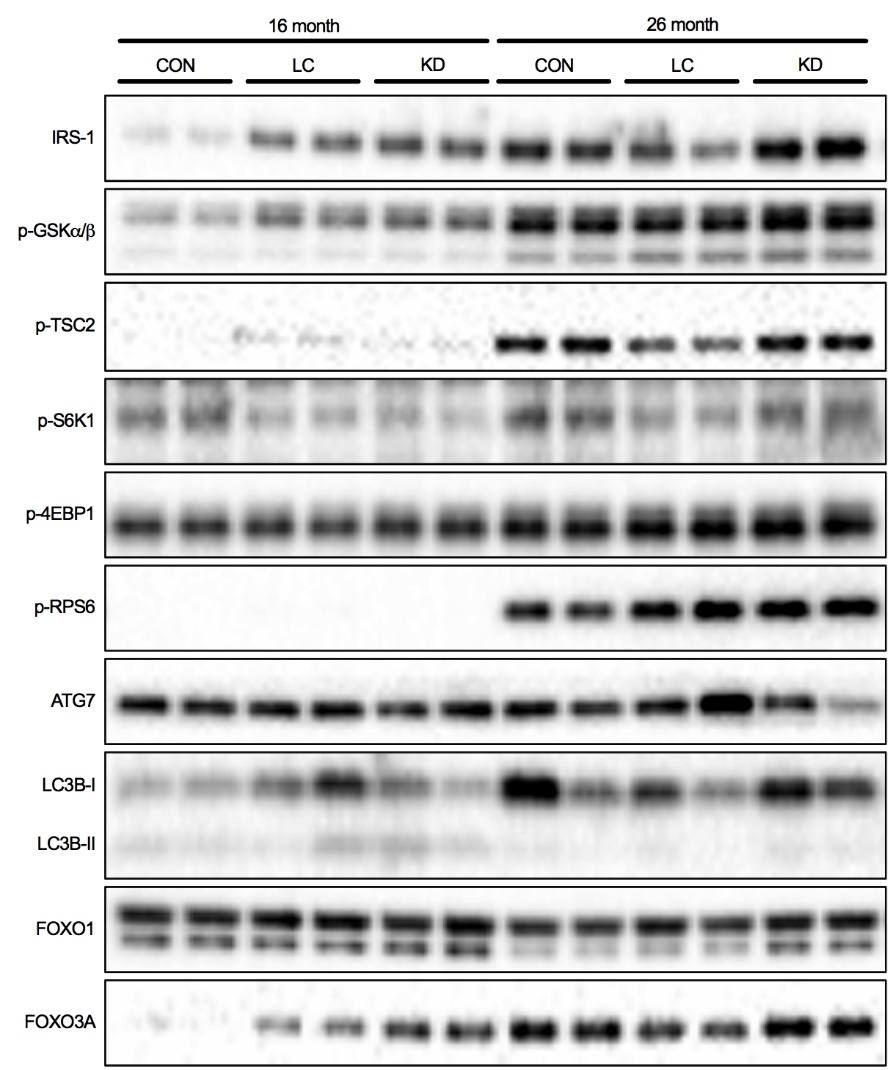


**Figure S4.** Complete western blot images of representative images illustrated in Figure 5C & F. Bands are from proteins extracted from the GTN of 16 and 26 month mice on a control (CON), low-carbohydrate (LC) and ketogenic diet (KD). Due to the exclusion of the LC group from the study, these images were edited to remove the LC group from the representative images.

**Supplementary References**

Baehr, L. M., West, D. W., Marcotte, G., Marshall, A. G., De Sousa, L. G., Baar, K., & Bodine, S. C. (2016). Age-related deficits in skeletal muscle recovery following disuse are associated with neuromuscular junction instability and ER stress, not impaired protein synthesis. Aging (Albany NY), 8(1), 127-146. doi:10.18632/aging.100879

Drake, J. C., Bruns, D. R., Peelor, F. F., 3rd, Biela, L. M., Miller, R. A., Miller, B. F., & Hamilton, K. L. (2015). Long-lived Snell dwarf mice display increased proteostatic mechanisms that are not dependent on decreased mTORC1 activity. Aging Cell, 14(3), 474-482. doi:10.1111/acel.12329

Drake, J. C., Peelor, F. F., 3rd, Biela, L. M., Watkins, M. K., Miller, R. A., Hamilton, K. L., & Miller, B. F. (2013). Assessment of mitochondrial biogenesis and mTORC1 signaling during chronic rapamycin feeding in male and female mice. J Gerontol A Biol Sci Med Sci, 68(12), 1493-1501. doi:10.1093/gerona/glt047

Gomes, A. V., Zong, C., Edmondson, R. D., Li, X., Stefani, E., Zhang, J., . . . Ping, P. (2006). Mapping the murine cardiac 26S proteasome complexes. Circ Res, 99(4), 362-371. doi:10.1161/01.RES.0000237386.98506.f7

Heinemeier, K. M., Olesen, J. L., Schjerling, P., Haddad, F., Langberg, H., Baldwin, K. M., & Kjaer, M. (2007). Short-term strength training and the expression of myostatin and IGF-I isoforms in rat muscle and tendon: differential effects of specific contraction types. J Appl Physiol (1985), 102(2), 573-581. doi:10.1152/japplphysiol.00866.2006

Hellerstein, M. K., & Neese, R. A. (1999). Mass isotopomer distribution analysis at eight years: theoretical, analytic, and experimental considerations. Am J Physiol, 276(6), E1146-1170. doi:10.1152/ajpendo.1999.276.6.E1146

Reid, J. J., Linden, M. A., Peelor, F. F., Miller, R. A., Hamilton, K. L., & Miller, B. F. (2020). Brain Protein Synthesis Rates in the UM-HET3 Mouse Following Treatment With Rapamycin or Rapamycin With Metformin. J Gerontol A Biol Sci Med Sci, 75(1), 40-49. doi:10.1093/gerona/glz069

Wadley, G. D., & McConell, G. K. (2007). Effect of nitric oxide synthase inhibition on mitochondrial biogenesis in rat skeletal muscle. J Appl Physiol (1985), 102(1), 314-320. doi:10.1152/japplphysiol.00549.2006
